# Perinatal asphyxia and Alzheimer's disease: is there a correlation?

**DOI:** 10.3389/fped.2025.1567719

**Published:** 2025-03-18

**Authors:** Bruna Petrucelli Arruda, Pamela Pinheiro Martins, Alexandre Hiroaki Kihara, Silvia Honda Takada

**Affiliations:** ^1^ Neurohistology Laboratory, Center for Computation, Mathematics and Cognition, Federal University of ABC, Sao Bernardo do Campo, São Paulo, Brazil; ^2^ Neurogenetics Laboratory, Center for Computation, Mathematics and Cognition, Federal University of ABC, Sao Bernardo do Campo, São Paulo, Brazil

**Keywords:** neonatal anoxia, hippocampus, development, neurodegenenerative diseases, hypoxic-ischemic encephalopathy

## Abstract

The perinatal development period is critical for the formation of brain structures responsible for cognitive functions. Disruptions during this phase, such as perinatal asphyxia, characterized by impaired gas exchange and hypoxia, can lead to long-lasting neuronal damage and increased susceptibility to neurodegenerative diseases, including Alzheimer's disease (AD). AD, the most common cause of dementia globally, is marked by amyloid plaques, neurofibrillary tangles, and progressive cognitive decline. Emerging evidence links perinatal asphyxia with an elevated risk of AD, highlighting the potential role of oxidative stress, neuroinflammation, and epigenetic modifications as mediators. This review explores the mechanisms underlying brain damage after perinatal asphyxia, emphasizing oxidative stress, inflammation, and epigenetic changes that contribute to lifelong neurodegenerative susceptibility. Additionally, biomarkers identified in animal models reveal parallels between perinatal asphyxia and AD pathology, such as amyloid precursor protein alterations, gliosis, and microglial activation. These findings suggest perinatal asphyxia may prime microglia and epigenetically alter gene expression, predisposing individuals to chronic neurodegeneration. Future research should leverage advanced methodologies, including transcriptomics, epigenomics, and aged brain organoid models, to elucidate early-life influences on AD development. Understanding these mechanisms may pave the way for novel prevention strategies targeting early-life risk factors for neurodegenerative diseases.

## 1 Introduction

The perinatal development period is of great significance for the formation of brain structures involved in cognitive functions. In recent years, a growing body of studies has demonstrated the crucial role of gestational factors in the development and functioning of the brain in postnatal life. Consequently, any alteration in this period can result in impairment of the neuronal network, which may manifest itself at different stages in postnatal life and predispose individuals to the development of diseases, including neurodegenerative diseases. In order to identify early prevention strategies, the identification of biomarkers is of particular significance and studies utilizing animal models to examine the impact of pathological conditions during development and throughout life are crucial.

Perinatal asphyxia represents a significant global health concern, characterized by impaired gas exchange and marked by extreme anoxia and hypercapnia. Prolonged or severe asphyxia can result in hypoxia and ischaemia. The resulting injury is diagnosed as hypoxic-ischemic encephalopathy (HIE) (1, 2), with an incidence of 1–8/1,000 live births in developed countries and 26/1,000 live births in developing countries The incidence of hypoxic-ischemic encephalopathy (HIE) is elevated in infants with low birth weight and prematurity, reaching 60% in such cases (3). HIE is a leading cause of developmental disabilities in children, accounting for 25% of such cases (4) These disabilities can manifest as learning difficulties, attention deficit hyperactivity disorder (ADHD), cerebral palsy, schizophrenia, and psychotic disorders (5).

In this context, the Developmental Origins of Health and Disease (DOHaD) hypothesis postulates that adaptive responses during development predispose the organism to adult disease. The developing nervous system is more susceptible to environmental factors that can permanently alter brain structure and function through epigenetic modifications, predisposing individuals to long-term pathologies such as cardiovascular disease (6, 7), metabolic syndrome (8) and depressive disorders (9). Recent studies have also suggested a possible link with neurodegenerative diseases (10, 11).

Neurodegenerative diseases represent a major threat to the health of the elderly population with a high impact on health and economic systems, and Alzheimer's disease (AD) is one of the most devastating. It accounts for 60%–70% of all cases of dementia, affecting approximately 44 million people worldwide (12), and with an aging population, the number of people with AD is estimated to triple by 2,050 (13), placing an increasing burden on healthcare systems.

Except for a small subset of familial cases, the causes of the vast majority of cases of Alzheimer's disease are unknown. Although its etiology is not fully understood, it is characterized by the formation of amyloid plaques and the aggregation of neurofibrillary tangles with dementia-causing mutations associated with its most insoluble proteins, β-amyloid peptide (Aβ) and microtubule-associated tau protein, whose accumulation leads to synaptic loss with subsequent neuronal atrophy, mainly affecting the medial temporal lobe, with progressive decline in cognitive function (14, 15). Studies suggest that the accumulation of β-amyloid plaques and reduction in hippocampal volume may precede the onset of clinical symptoms (16–18), and that loss of neuronal function in humans can be detected decades before the onset of AD (19).

Despite the lack of knowledge about the cause, environmental factors appear to play an important role in the development of AD, such as cerebral infarction (20), sleep-disordered breathing (21) and hypoxia (22), and the accumulated research to date suggests an association between perinatal asphyxia and an increased risk of developing AD (23, 24).

The mechanisms by which AD develops following neonatal hypoxia are unknown, but in this review we will focus on how perinatal asphyxia and its key events may contribute to the development of AD in later life.

## 2 Brain damage after perinatal asphyxia

During pregnancy, cerebral blood flow provides oxygen and glucose to the fetal brain, maintaining homeostasis. Importantly, is also provides the distribution of hormones that are essential for proper neurodevelopment, such as thyroid hormones (25), glucocorticoids (26), neurosteroids [estrogen, progesterone, allopregnanolone, testosterone and others (27, 28)]. Perinatal asphyxia, especially resultant from placental disorders, can interrupt or impair the transfer of these hormones to the fetus and, consequently, lead to neurodevelopmental alterations. These conditions result in a reduced supply of oxygen and glucose, which triggers a continuous temporal injury process consisting of 4 phases.

The primary phase occurs during oxygen and glucose deprivation, which triggers an energy failure with membrane depolarization and cellular edema. This is followed by increased release of excitatory neurotransmitters such as glutamate into the synaptic cleft, where they accumulate due to impaired energy-dependent reuptake, leading to a greater influx of cellular Ca²+ (29). As a result, neurotoxic cascades are triggered, causing oxidative stress, inflammation, cell death by necrosis and activation of intracellular apoptotic signaling cascades (30).

This is followed by a latent phase lasting 6–12 h, characterized by recovery of cerebral metabolism, inflammation and the continuation of apoptotic cascades (31, 32). If the primary lesions are moderate or severe, this transient recovery is followed by a second energy failure accompanied by seizures, secondary cytotoxic edema, neuroinflammation and programmed cell death.

Considering this whole process, some events may persist throughout life, such as sensitization to inflammation, increased susceptibility to seizures, long-lasting inflammation and gliosis, impaired maturation and myelination, altered proliferation and synaptogenesis, and epigenetic modifications. This phase is called the tertiary phase and is characterized by damage to brain plasticity and connectivity that persists throughout life (33).

In this cascade, two important events in the development of pathologies stand out and possibly contribute to the prolonged neurodegeneration that could lead to the association with Alzheimer's disease: oxidative stress and inflammation, both of which interact with each other. Once activated, many cells in the immune system generate free radicals, and the overproduction of free radicals also induces an inflammatory response.

**Figure 1:**
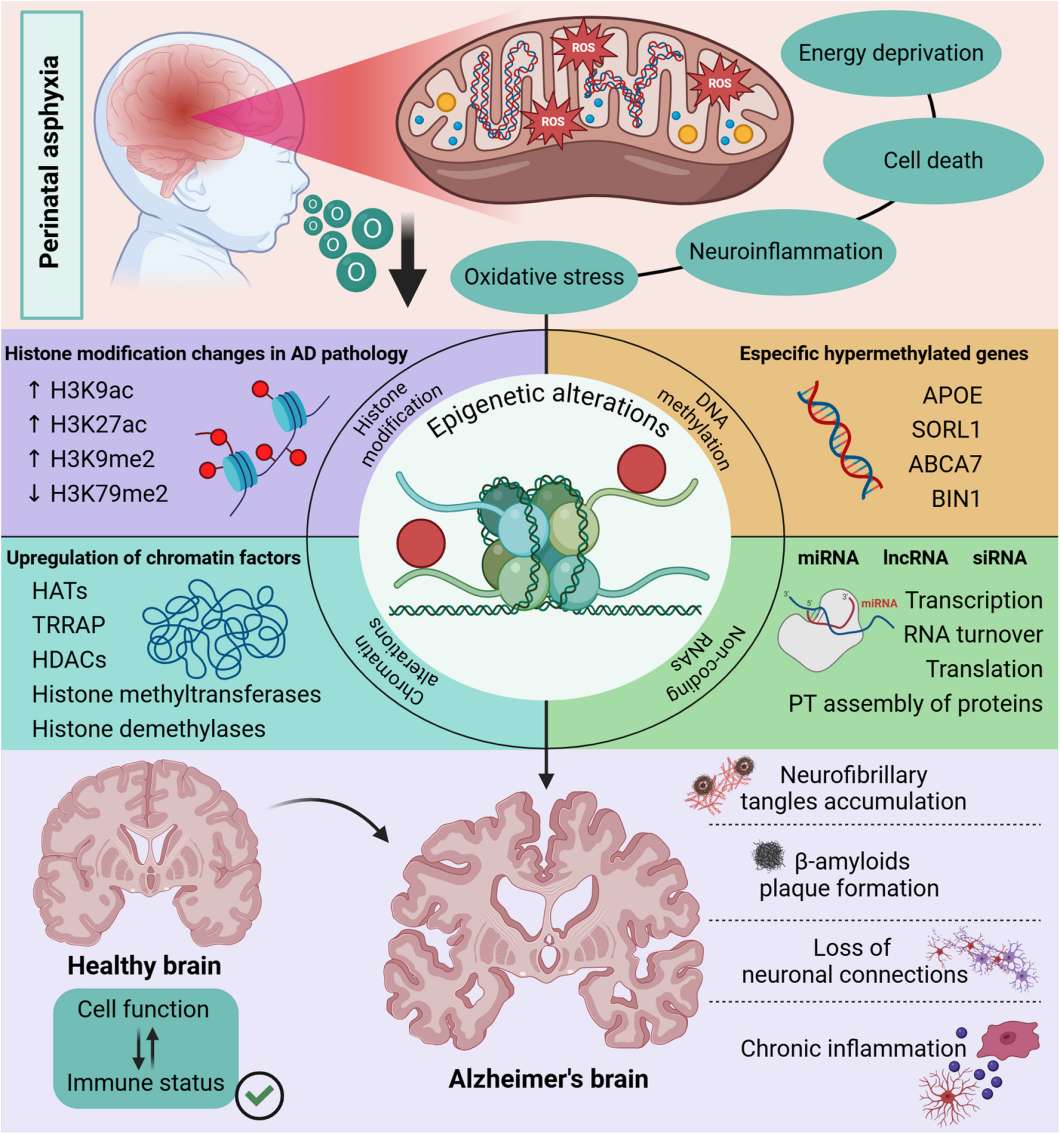
Epigenetic changes in the Alzheimer’s brain. The neuronal damage caused by perinatal asphyxia is characterized by mitochondrial dysfunction since anabolic pathways that lead to oxidative stress are activated. Interestingly, studies have shown the influence of mitochondrial alteration on epigenetic regulation in DNA methylation mechanisms, expression of proteins that control the function of histones and molecules with an important role in post-transcriptional modifications, such as ncRNAs. These epigenetic modifications linked to the functioning of clearance pathways and cell function have a direct impact on the clinical signs observed in patients with Alzheimer’s disease. Created in BioRender. Kihara A (2025), https://BioRender.com/q55k045.

The epigenetic modifications (Figure 1), such as changes in histone proteins making DNA more or less accessible for gene expression, as well as changes in chromatin due to upregulation of factors that control acetylation and methylation, are also strong candidates to explain the correlation between perinatal asphyxia and Alzheimer's disease (34). In addition, studies have shown that in Alzheimer's disease there are alterations in DNA methylation in specific genes such as APOE, an apolipoprotein that is reduced in the hippocampus of patients diagnosed with Alzheimer's and is related to increased degradation of amyloidogenic Aβ. As well as SORL 1, which is also reduced in patients and plays a fundamental role in APP turnover, but in its absence or at insufficient levels, APP is cleaved into products that form amyloidogenic Aβ (35–37).

### 2.1 Neuroinflammation and oxidative stress

Even after secondary cell death, the immune response is harnessed and both microglia and astrocytes are activated by pro-inflammatory mediators such as cytokines, reactive oxygen species and debris from damaged cells. Thus, information in the central nervous system is characterized by the classical activation of microglia (M1) and the production of cytokines such as TNF-α, IL1-β, IL-6, IL-8 and ROS. This is followed by alternative activation (M2), which leads to the release of anti-inflammatory cytokines such as IL-4 and IL-3, resulting in the removal of ROS and tissue repair. The mechanisms responsible for regulating microglial phenotypes in the neonatal CNS are poorly understood and this M1/M2 interaction does not appear to follow a strict differentiation; depending on signaling, phenotypes may switch from M1 to M2 or vice versa during lesion progression (38, 39).

In fact, microglia are one of the main mediators of the inflammatory response after hypoxic-ischemic injury, and their activation and aggregation are markers of HIE in human neonates. A retrospective study of 178 brains from post-mortem neonates showed that those diagnosed with HIE who died had a marked infiltration of microglia in the region of the dentate gyrus of the hippocampus, compared with infants who died from other causes and had lower numbers of microglial cells (40). Another clinical study described an increase in potentially neurotoxic inflammatory factors in microglia (galectin-3 and MMP-9) in babies with HIE (41).

In addition, microglia crosstalk with astrocytes, another important cell population with multiple roles, including regulating blood flow, increasing the supply of energy metabolites and maintaining the extracellular balance of fluids and neurotransmitters (42), as well as participating in the immune response. In this context, microglial activation and the cytokines it produces trigger astrocyte activation. Astrocytes in turn hypertrophy, increase glial fibrillary acid expression and form a glial scar around the affected area (43). Astrocytes also produce and release pro-inflammatory cytokines and chemokines that exacerbate brain damage.

Clinical studies show that these cytokines and other inflammatory mediators released by glial cells, such as TNF-α, IL1-β, IL-6, IL-8 and IL-10, are significantly increased in the serum and cerebrospinal fluid (CSF) of asphyxiated neonates (44–47). In addition, the release of TNF-α, IL1-β and IL-6 promotes increased synthesis of other cytokines and adhesion molecules (48), and together they induce the production and secretion of mediators such as ROS, leading to cell damage and death (49).

During asphyxia, several mechanisms are responsible for oxidative stress, including an increase in Ca²+ influx, which activates nitric oxide (NO) synthase, generating NO free radicals that contribute to the release of ROS, a reduction in the components of the electron transport chain, and an increase in iron release from ferritin and increased ATP degradation (50).

In addition, studies suggest that oxidative stress plays a role in the crosstalk between inflammatory systems (51, 52). The rapid increase in the levels of pro-inflammatory cytokines secreted by glial cells leads to their accumulation in the brain tissue, resulting in direct injury by further increasing the levels of toxic NO.

These processes result in an accumulation of ROS, which cannot be eliminated immediately by antioxidant enzymes due to disrupted metabolism, and cause damage to lipids, proteins and nucleic acids, leading to lipid and protein oxidation and degeneration of deoxyribonucleic acid (DNA) (53), inducing neuronal apoptosis and inhibiting neurogenesis, resulting in damage later in life.

## 3 Biomarkers in brain damage after perinatal asphyxia and Alzheimer's disease

In recent years, a significant number of studies have focused on identifying factors that may link the effects of perinatal asphyxia to the risk of developing neurodegenerative diseases such as AD.

Since 1997, researchers have demonstrated delayed neuronal death following perinatal asphyxia (54). One study found significant changes in amyloid precursor protein levels at P30 and in the expression of metallopeptidases involved in amyloid metabolism at P10 in rats exposed to prenatal hypoxia (55).

The researchers found that PSA-NCAM is a neuropathological marker of neurodegeneration that is evident as early as 8 days after neonatal hypoxia-ischemia, as the abnormal accumulation of PSA-NCAM preceded the accumulation of phospho^Ser396^ tau observed 30 days after injury. Furthermore, a study in guinea pigs provided the first evidence of neurodegeneration, finding neuronal loss, gliosis, and monoaminergic and cholinergic alterations 3 months after perinatal asphyxia (56).

In an experimental model of newborn pigs subjected to perinatal asphyxia, it has been shown that the injury reduces levels of beta-amyloid (1–42) in cerebrospinal fluid (57). In fact, a decrease in CSF AB42 is the first biomarker change to occur in Alzheimer's disease.

In a model of prenatal hypoxia, transgenic mice showed deficits in memory and spatial learning, a significant reduction in synapses and increased levels of amyloid precursor protein (24). Gabaergic inhibitory interneurons parvalbumin (PV) and calbindin-1 (Calb1), whose interaction with hippocampal pyramidal cells is fundamental to memory and learning processes, are also affected late after neonatal HI (58).

In APPSwe/PS1A246E transgenic mice, perinatal hypoxia also exacerbated cognitive impairment observed in the Morris water maze test and AD pathology by affecting synaptic ultrastructure, increasing senile plaque and Aβ peptide production, and exacerbating astrocyte and microglial activation in adult animals (59). A mouse model of HI also showed prolonged gliosis that correlated with a late deficit of Calb1 (60).

In humans, one study found high levels of the β-amyloid 1–38 and β-amyloid 1–42 peptides in the blood of babies up to 15 days after perinatal asphyxia. In contrast, levels of β-amyloid peptide 1–40 were lower between 8 and 14 days after asphyxia, and TAU protein increased in the first 7 days, decreased between 8 and 14 days and increased again up to 15 days after injury (61).

Neuroinflammation plays a central role in the pathogenesis of AD, and several studies have demonstrated the involvement of glial cells in the production of pro-inflammatory mediators that contribute to the exacerbation of neurodegeneration [for review, see (62)]. In addition to the well-known astrogliosis present in AD (63) and other neurodegenerative diseases, there is increasing evidence for the involvement of microglia as protagonists in this process (64–66), and a cell type of microglia with a specific transcriptional signature, called “disease-associated microglia” (DAM), has been identified, whose functions are still undetermined, but which are present in AD (67).

Recently, the hypothesis that microglial priming predisposes individuals to neurodegenerative disease has gained prominence and is being better investigated (68–70). Microglial priming corresponds to an enhanced response of microglial activation to a second inflammatory stimulus (71). Thus, microglial activation during development renders these cells susceptible to subsequent infection or systemic inflammation (68), contributing to the progression of chronic neurodegenerative diseases (71).

Furthermore, since gene expression patterns reflect aging and neurodegeneration, and studies have shown that several genes in the human cortex change their expression during fetal and postnatal development, it is reasonable to expect that perinatal factors influence these processes. An important gene during development that is altered in adult neurogenesis and implicated in Alzheimer's disease is the RE1 accelerator transcription factor, REST (72). This factor is induced by hypoxia and leads to changes in about 20% of hypoxia-suppressed genes (73). Overexpression of presenilin genes related to y-secretase has also been demonstrated in lymphocytes 15 days after perinatal asphyxia (74) and increased expression of these genes correlates positively with findings in patients with neurodegenerative disease (75).

## 4 Conclusions and future perspectives

The research accumulated so far shows changes similar to those found in the pathology of Alzheimer's disease after perinatal asphyxia, suggesting the latter as a risk factor for Alzheimer's disease. These findings open the door to hypotheses such as the existence of microglial priming, epigenetic alterations and the DOHaD theory. However, further experiments are needed to prove these theories and to understand the mechanisms by which events in early life influence the development of neurodegenerative diseases.

Advances in methodologies that could be applied in the neurosciences field, such as single-cell transcriptome, epigenome and even the development of strategies to use aged brain organoids might be promising tools to elucidate the impact of early life events in neurodegenerative diseases, as well as the use of transgenic and non-transgenic mice, such as the Senescence Accelerated Mouse-Prone 8 (SAMP8) mice, and other animal models that mimic the neurodegenerative diseases phenotype.
